# Anaerobic animals from an ancient, anoxic ecological niche

**DOI:** 10.1186/1741-7007-8-32

**Published:** 2010-04-06

**Authors:** Marek Mentel, William Martin

**Affiliations:** 1 Department of Biochemistry, Faculty of Natural Sciences, Comenius University, Mlynská dolina CH-1, 842 15 Bratislava, Slovakia; 2 Institute of Botany III, University of Düsseldorf, 40225 Düsseldorf, Germany

## Abstract

Tiny marine animals that complete their life cycle in the total absence of light and oxygen are reported by Roberto Danovaro and colleagues in this issue of *BMC Biology*. These fascinating animals are new members of the phylum Loricifera and possess mitochondria that in electron micrographs look very much like hydrogenosomes, the H_2_-producing mitochondria found among several unicellular eukaryotic lineages. The discovery of metazoan life in a permanently anoxic and sulphidic environment provides a glimpse of what a good part of Earth's past ecology might have been like in 'Canfield oceans', before the rise of deep marine oxygen levels and the appearance of the first large animals in the fossil record roughly 550-600 million years ago. The findings underscore the evolutionary significance of anaerobic deep sea environments and the anaerobic lifestyle among mitochondrion-bearing cells. They also testify that a fuller understanding of eukaryotic and metazoan evolution will come from the study of modern anoxic and hypoxic habitats.

## Commentary

The newly reported tiny marine animals that complete their life cycle in the total absence of light and oxygen are members of the phylum Loricifera, a phylum discovered less than 30 years ago, and they are less than a millimetre in size [1]. They were collected from a deep basin at the bottom of the Mediterranean Sea, where they inhabit a nearly salt-saturated brine that, because of its density (>1.2 g/cm^3^), does not mix with the waters above. As a consequence, this environment is completely anoxic and, due to the activity of sulphate reducers, contains sulphide at a concentration of 2.9 mM. Despite such harsh conditions, this anoxic and sulphidic environment is teeming with microbial life, both chemosynthetic prokaryotes that are primary producers [2], and a broad diversity of eukaryotic heterotrophs at the next trophic level [3,4]. That this ecological niche also supports animals is a surprise that poses all sorts of interesting questions. Despite being unexpected, however, the finding ties together recent developments from several independent fields (marine biology, cell biology, evolutionary theory and geochemistry) that all point to the evolutionary significance of eukaryotic life in anaerobic environments.

The first question raised is 'how?'. How is it possible that animals can inhabit this anoxic and sulphidic environment? This might seem impossible to some, after all one often reads that 'animals have an absolute requirement for oxygen' [5] or 'sulphide is poisonous' [6]. However, not all animals are strictly dependent upon oxygen. Some use different terminal electron acceptors other than oxygen in their mitochondrial respiratory chains, most commonly fumarate, leading to the excretion of succinate and propionate [7], often accompanied by acetate excretion as well [8]. Since the mechanism of sulphide toxicity to animals entails the inhibition of cytochrome *c *oxidase [9], mitochondria that are not dependent upon that final mediator of an electron transfer to O_2 _also do not have such a problem with sulphide. Among animal lineages, facultative anaerobic mitochondria have been studied from various free living invertebrates, including the oyster *Mytilus *(Mollusca) [10], the peanut worm *Sipunculus *(Sipuncula) [11] or the polychaete worm *Arenicola *(Annelida) [12] and parasites like *Fasciola *(Platyhelminthes) [13] and *Ascaris *(Nematoda) [14]. However, such oxygen-independent energy metabolism in animals is often restricted to some stages -- albeit sometimes prolonged -- of the lifecycle. The Loriciferans that Danovaro *et al. *[1] describe spend their entire life cycle in the sediment: what was once seen as an 'absolute requirement' for O_2 _among animals should now be seen as a lineage-specific preference, albeit it one that is very pronounced, especially among those animals that, like ourselves, live on land, permanently above the soil line.

### Anaerobic mitochondria: more common all the time

**Figure 1:**
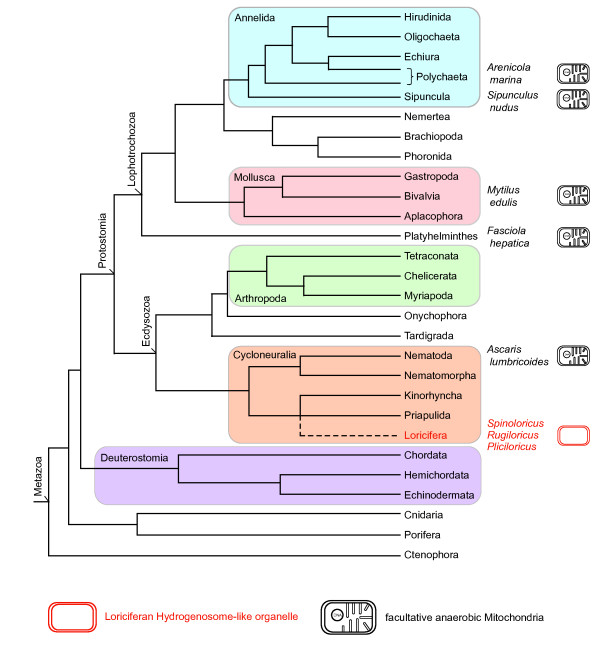
**Schematic phylogeny of animals based on the report by Dunn *et al ***[48]** which, however, did not include the phylum Loricifera **[1]** (highlighted in red)**. It was placed here (dotted line) as branching with Priapulida and Kinorhyncha as reported elsewhere [49,50]. Higher taxon designations are those used in references [48-50]. The Cycloneuralia, where Loricifera belong, are currently grouped within the Ecdysozoa, which also includes nematodes and insects. To the right of the phylogeny, several species are listed whose facultatively anaerobic mitochondria have been studied [10-14]; in no way does this imply the absence of anaerobic mitochondira in other groups. No biochemical data are yet available for the Loriciferan mitochondria [1].

A look at the phylogenetic distribution of characterized anaerobic mitochondria among animal lineages shows that these are not clustered but spread across metazoan phylogeny (Figure 1). Are these recent adaptations to anaerobic habitats or are they holdovers from our more distant evolutionary past? The biochemistry and the enzyme equipment used in the facultatively anaerobic mitochondria of metazoans is nearly identical across lineages [7,10-15], strongly indicating a common origin from the metazoan ancestor that might have lived some 600 million years (MY) ago [16]. The examples in Figure 1 cover both the Lophotrochozoa and the Ecdysozoa, where the newly-described Loricifera with their distinctive organelles belong. Although no biochemical data are yet available for these intriguing new Loriciferan mitochondria, from the electron micrographs presented by Danovaro *et al. *[1] the organelles look like hydrogenosomes - anaerobic forms of mitochondria that generate H_2 _and adenosine triphosphate (ATP) from pyruvate oxidation [17] and which were previously found only in unicellular eukaryotes. Danovaro *et al. *[1] also report that the animals harbour structures resembling prokaryotic endosymbionts, reminiscent of the methanogenic endosymbionts found in some hydrogenosome-bearing protists; fluorescence of F_420_, a typical methanogen cofactor [18], or lack thereof, will bring more insights as to what these structures are.

**Figure 2:**
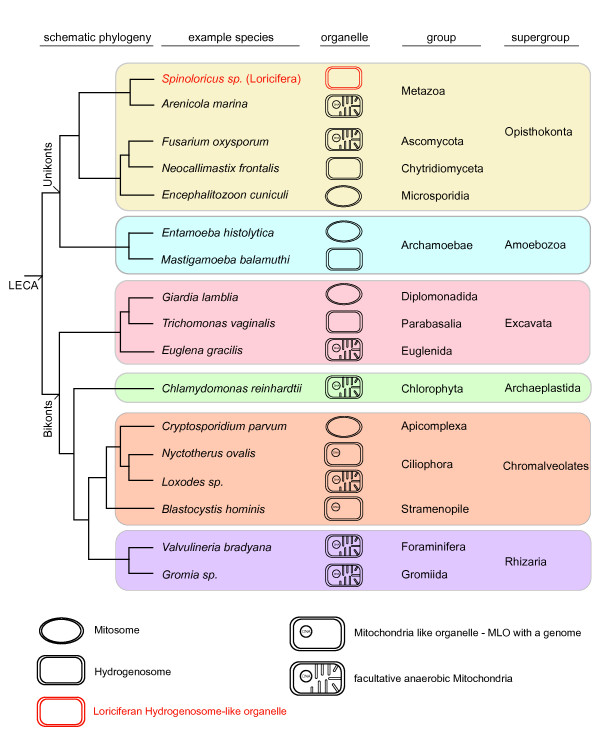
**Protistan schematic phylogeny representing current view about eukaryotes phylogeny grouping all known eukaryotic life to six major clades or supergroups **[20]**, together with information about the mitochondria from some anaerobic, facultatively anaerobic or parasitic representatives **[19,21]. With greater sampling of anoxic habitats [3,4,27], additional information about anaerobic eukaryotes can be expected. The latest additions are the new species of loriciferan animals (red) from the anoxic L'Atalante basin [1]. LECA: last eukaryotic common ancestor. Note the absence of primitively amitochondriate lineages among eukaryotes [19,21].

If we follow the anaerobic lifestyle further back into evolutionary history (Figure 2), beyond the origin of the metazoans, we see that the phylogenetic distribution of eukaryotes with facultative anaerobic mitochondria, eukaryotes with hydrogenosomes and eukaryotes that possess mitosomes (reduced forms of mitochondria with no direct role in ATP synthesis [19]) shows a similar picture to that seen for animals. In all six of the major lineages (or supergroups) of eukaryotes that are currently recognized [20], forms with anaerobic mitochondria have been found [21]. The newest additions to the growing collection of anaerobic mitochondrial metabolisms are the denitrifying foraminiferans [22,23]. For this group, the underlying enzymes also have yet to be worked out. However, for the remainder of the eukaryotes summarized in Figure 2, a handful of about a dozen enzymes make the difference between a 'normal' (in the sense of 'familiar from older college textbooks') O_2_-respiring mitochondrion found in mammals, and the energy metabolism of eukaryotes with anaerobic mitochondria, hydrogenosomes or mitosomes [7,19,21,24]. Notably, the full complement of those enzymes, once thought to be specific to eukaryotic anaerobes, surprisingly turned up in the green alga *Chlamydomonas reinhardtii *[24], which produces O_2 _in the light, has typical O_2_-respiring mitochondria but, within about 30 min of exposure to heterotrophic, anoxic and dark conditions, expresses its anaerobic biochemistry to make H_2 _[25,26] in the same way as trichomonads, the group in which hydrogenosomes were discovered [17]. Thus, *Chlamydomonas *provides evidence which indicates that the ability to inhabit oxygen-harbouring, as well as anoxic environments, is an ancestral feature of eukaryotes and their mitochondria; in that sense it is a true missing link that unites mitochondria like our own and those from the anoxic world [19].

It is not yet known what role, if any, the mitochondria of the newly discovered loriciferans play in the energy metabolism of those animals and, unfortunately, the same is true for the myriad of fascinating eukaryotic protists that inhabit the very same environment where the anoxic loriciferans were found. In a series of recent papers, Stoeck and colleagues have surveyed the protistan diversity from L'Atalante and similar habitats with environmental sequencing and metagenomic techniques [3,4,27]. Those reports lead to a wealth of representatives from many of the same groups shown in Figure 2 being uncovered: ciliates, fungi and chromalveolates, in addition to representatives of many other eukaryotic groups, including the choanoflagellates [3], which are regarded as the unicellular sisters to the metazoan clade. The investigation of anaerobic mitochondria remains an area of rapid progress and it will be a challenge to discover what those mitochondria are doing in real-life anoxic and hypoxic environments, which are very widespread among modern habitats [28] and where eukaryotic anaerobes abound [27].

### Evolutionary significance?

What is the evolutionary significance of the new findings? The L'Atalante basin has been anoxic for only about 50,000 years [1] but have all of its microbial and metazoan inhabitants only recently adapted to life in anoxic conditions during that time? Hardly. Nobody seriously considers that anaerobic *prokaryotes *dwelling in such anaerobic habitats, such as methanogens and sulphate reducers, have only recently adapted to anaerobic niches. The prokaryote inhabitants have existed for well over a billion years, and have reached this new habitat by dispersal, not by adaptive evolution *de novo *and *in situ*. Indeed, geochemical evidence has shown that methanogenesis and sulphate reduction, and the niches in which they occur, are truly ancient [29,30]. (For marine environments, dispersal is not a fundamental problem because, despite vast distances, similar types of seafloor habitats often harbour similar communities, from microbes to large animals [31]). However, when it comes to *eukaryotes*, there is still a curious tendency to assume that eukaryotes only invaded anaerobic niches of late. Perhaps this stems from a tendency (latently anthropocentric, no doubt) to view mitochondria as obligately O_2_-dependent organelles with the anaerobic forms of mitochondria being rare exceptions, even though the data (for example in Figures 1 and 2 or elsewhere [7,8,17,19,21]) tell us otherwise. In environments such as the L'Atalante basin, 'normal', O_2_-dependent mitochondria are the rare exception, if they exist at all, but eukaryotes abound [1,3,4,32,33]. Hence, further study of mitochondria from such environments should be revealing. That, however, is easier said than done, since, as seen in the present study [1] as in other work on organisms from anoxic marine sediments [32], a considerable effort has to be invested in order just to demonstrate that the organisms are even alive and not just sunken carcasses. Work on organisms from these environments poses substantial technical challenges, making every new insight all the more exciting.

While Danovaro *et al. *opt for the term 'enigmatic' in discussing the evolutionary significance of their findings, we have a decidedly different view. Given that anaerobic forms of mitochondria are widespread throughout the eukaryotic world, we see eukaryotes in anaerobic habitats as evidence for evolution in the Darwinian sense of descent with modification, with the traits that support survival in anaerobic environments having been conserved from earlier phases of Earth's history. This view is underpinned by what geologists and geochemists have been trying to tell biologists over the last 10 years about the prevalence of anoxic and sulphidic environments during the early phase of eukaryotic and metazoan evolution, but with the biologists perhaps not taking as much notice as they should.

### The bigger picture: add geological time

What are the geologists trying to tell us about anoxic and sulphidic marine habitats? A readable summary of about 10 year's progress is given in three papers [34-36]. In a nutshell, the geologists are saying that the rise in atmospheric oxygen some 2.4 billion years ago is one thing, but that the oxygen levels in the ocean, where evolution was taking place, is quite another. Several lines of isotope evidence indicate that deep ocean waters were not fully oxygenated until about 580 MY ago, about the time when the first large animals made their fossil debut. The reason for this anoxia, they say, has to do with high levels of sulphide in marine environments, from the workings of sulphate reducers [36], themselves strict anaerobes. The message for biologists is that the Earth's oceans appear to have been largely anoxic and sulphidic (like the L'Atalante basin, but not hypersaline) below the photic zone (the upper about 200 m) [34,36] and, possibly, also in the lower photic zone [35] for the time spanning roughly 1.8 billion years ago to about the beginning of the Cambrian period 560 MY ago. That was the time during which eukaryotes arose and diversified [21]. Hence, it should hardly be surprising that the anaerobic lifestyle is widespread among eukaryotic lineages, right up into the animals [37]. One view is that the oxygenation of deep environments allowed the animals to become larger [38], which is different from saying that oxygen might in any way be causal to the Cambrian appearance of diverse animal forms [39]. Geologists have not been trying to hide their perspective from us biologists [40-42], nor have we biologists been trying to hide our progress in understanding eukaryotic anaerobes [21,43-46]. However, it seems that it will still take some time for a new default view of deep marine environments in the Late Precambrian -- a new synthesis of sorts -- to be accepted by biologists. The scenario is one of widespread anoxic and sulphidic habitats, almost certainly (in our view) teeming with little eukaryotic creatures, all with their mitochondria well suited to life with little or no oxygen [37] and all more or less like the ones we see in anoxic and sulphidic environments today. Such environments staged and witnessed the origin of evolution's greatest early inventions [47] and the new insights emerging from them deserve our close attention.

## Abbreviations

ATP: adenosine triphosphate; MY: million years.
